# Sagittal instability with inversion is important to evaluate after syndesmosis injury and repair: a cadaveric robotic study

**DOI:** 10.1186/s40634-020-00234-w

**Published:** 2020-03-30

**Authors:** Neel K. Patel, Conor I. Murphy, Thomas R. Pfeiffer, Jan-Hendrik Naendrup, Jason P. Zlotnicki, Richard E. Debski, MaCalus V. Hogan, Volker Musahl

**Affiliations:** 1 Orthopaedic Robotics Laboratory, Department of Orthopaedic Surgery, Department of Bioengineering, University of Pittsburgh, 300 Technology Drive, Pittsburgh, PA 15219 USA; 2 Department of Trauma and Orthopaedic Surgery, Witten/Herdecke University, Cologne Merheim Medical Centre, Ostmerheimer Strasse 200, 51109 Köln, Germany; 3 Department of Orthopaedic Surgery, Center for Sports Medicine, University of Pittsburgh, 3200 S Water Street, Pittsburgh, PA 15203 USA

**Keywords:** Ankle syndesmosis, Tricortical screw, Suture button, Distal tibiofibular kinematics

## Abstract

**Purpose:**

Disruption of the syndesmosis, the anterior-inferior tibiofibular ligament (AITFL), the posterior-inferior tibiofibular ligament (PITFL), and the interosseous membrane (IOM), leads to residual symptoms after an ankle injury. The objective of this study was to quantify tibiofibular joint motion with isolated AITFL- and complete syndesmotic injury and with syndesmotic screw vs. suture button repair compared to the intact ankle.

**Methods:**

Nine fresh-frozen human cadaveric specimens (mean age 60 yrs.; range 38–73 yrs.) were tested using a six degree-of-freedom robotic testing system and three-dimensional tibiofibular motion was quantified using an optical tracking system. A 5 Nm inversion moment was applied to the ankle at 0°, 15°, and 30° plantarflexion, and 10° dorsiflexion. Outcome measures included fibular medial-lateral translation, anterior-posterior translation, and external rotation in each ankle state: 1) intact ankle, 2) AITFL transected (isolated AITFL injury), 3) AITFL, PITFL, and IOM transected (complete injury), 4) tricortical screw fixation, and 5) suture button repair.

**Results:**

Both isolated AITFL and complete injury caused significant increases in fibular posterior translation at 15° and 30° plantarflexion compared to the intact ankle (*p* < 0.05). Tricortical screw fixation restored the intact ankle tibiofibular kinematics in all planes. Suture button repair resulted in 3.7 mm, 3.8 mm, and 2.9 mm more posterior translation of the fibula compared to the intact ankle at 30° and 15° plantarflexion and 0° flexion, respectively (*p* < 0.05).

**Conclusion:**

Ankle instability is similar after both isolated AITFL and complete syndesmosis injury and persists after suture button fixation in the sagittal plane in response an inversion stress. Sagittal instability with ankle inversion should be considered when treating patients with isolated AITFL syndesmosis injuries and after suture button fixation.

**Level of evidence:**

Controlled laboratory study, Level V.

## Background

Syndesmotic ankle injuries are a relatively common injury, reported in up to 18% of ankle sprains and 23% of all ankle fractures [14, 30]. Disruption of the anterior inferior tibiofibular ligament (AITFL), posterior inferior tibiofibular ligament (PITFL), and interosseous membrane (IOM) is a predictor of worse outcomes after ankle injury [32]. Poor long-term patient outcomes after syndesmotic injury are thought to be due to tibiofibular instability, which create altered ankle kinematics resulting in accelerated posttraumatic ankle arthritis [12, 26]. The current treatment algorithm for syndesmotic injuries is based on the tibiofibular clear space widening on anterior-posterior or mortise ankle radiograph with an external rotation stress test under fluoroscopy or with application of lateral traction to the fibula intraoperatively [13, 24].

The West Point Ankle grading system (I-III) is often used to make treatment decisions regarding syndesmotic injuries [8]. Grade I injuries, partial tears of the AITFL, are considered to be stable injuries and are typically treated conservatively [23, 29]. Grade III injuries involve disruption of the complete syndesmosis complex, including the AITFL, PITFL, and IOM, and are usually treated surgically with either cortical screw or a suture button fixation [1]. However, the optimal syndesmotic fixation method to restore syndesmotic kinematics remains unclear due to conflicting and variable results of previous studies [3, 15, 25]. Management of a grade II injury, which involves a complete isolated disruption of the AITFL, is more controversial with regard to the need for surgical fixation depending on the instability of the distal tibiofibular joint [18]. There is still an ongoing controversy about how the AITFL contributes to fibular stability [4, 21, 22]. Though a previous study has shown that ankle instability with inversion stress is important to consider, this has not been commonly investigated since an external rotation stress to the ankle is the primary mechanism of syndesmosis injury [28].

Thus, the objective of this study was to quantify the effects of isolated AITFL and complete syndesmotic injury as well as syndesmotic screw and suture button fixation on tibiofibular joint motion in response to inversion stress. It was hypothesized that 1) complete syndesmosis injury would lead to significantly increased tibiofibular joint motion when compared to isolated AITFL injury; and that 2) screw fixation would restore tibiofibular joint motion closer to the intact when compared to suture button repair.

## Methods

Nine fresh-frozen human cadaveric tibial plateau-to-toe specimens with a mean age of 60 years (range 38–73 years) were tested using a 6 degree-of-freedom (DOF) robotic testing system (MJT Model FRS2010, Chino, Japan). Specimens were store at − 20 °C and thawed at room temperature for 24 h prior to testing. Each specimen was examined manually and radiologically before testing to exclude specimens with previous ligamentous injuries, fibula fractures, osteoarthritis, or other bony abnormalities. The AITFL, PITFL, and IOM were visualized by creating a 10-cm skin incision along the posterolateral aspect of the fibula and dissecting superficially along the anterior and posterior borders of the fibula. A 4-hole one-third tubular plate was secured to the distal fibula with the second most distal hole approximately 2 cm above the plafond to standardize the placement of the tricortical screw and suture button during syndesmotic fixation. The hole for the tricortical screw and suture button was predrilled in the intact ankle with the foot in 0° flexion to avoid malreduction of the fibula after transection of the syndesmotic ligaments.

**Fig. 1:**
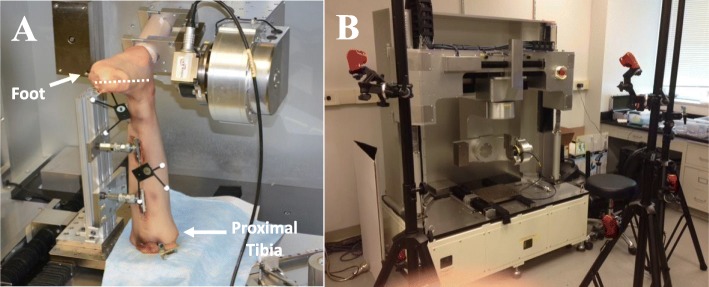
**a** Experimental setup with full length fibula specimen rigidly mounted to the robotic testing system through the calcaneus and a universal force-moment sensor (UFS). Optical motion capture markers are noted on the fibula and tibia. The dashed white line represent the axis of rotation of for inversion as defined by the middle of the talus and the 2nd metatarsal. **b** The experimental setup with the robotic testing system surrounded by six Motion Capture Cameras in a semicircular configuration

A minimal anterior arthrotomy was performed for visualization of the anterior aspect of the talus and the subtalar joint was fixed with two wood screws under fluoroscopic guidance. Fixation of the subtalar joint was necessary in order to precisely control tibiotalar joint motion and apply loads and torques in a repeatable manner. The skin and subcutaneous tissues overlying the posterior calcaneus were removed, allowing it to be potted in an epoxy compound (Bondo, 3 M, St. Paul, MN). The potting material, which is an extension of the posterior aspect of the calcaneus, was then secured within a custom-made aluminum clamp and rigidly fixed to the upper end plate of the robotic manipulator through a universal force/moment sensor (UFS, ATI Delta IP60 (SI-660-60), Apex, NC). The tibia was rigidly mounted to the lower plate of the robotic testing system using a L-bracket attached to the base plate of the robotic testing system and screws through the bracket in the proximal tibia. In this construct, the full length of the fibula was maintained and fibular motion was unconstrained (Fig. 1a). During the experimental protocol, the specimen was kept moist with saline.

An optical motion capture marker triad was mounted to the distal fibula and distal tibia (Fig. 1a). Six 1280 × 1024 240 Hz Motion Capture Cameras (Optitrack Flex 13, Corvallis, OR) were positioned in a semicircle around the robotic testing system to detect the optical markers attached to the tibia and fibula (Fig. 1b). A digitizer was used to define anatomic landmarks including the tibial tuberosity, Gerdy’s tubercle, the tibiotalar joint center, and the lateral malleolus to create coordinate systems for the tibia and fibula [11]. The tibia’s proximal-distal axis was defined as the vector from the tibiotalar joint center to the tibial tuberosity. The medial-lateral axis of the tibia was defined as the vector from the tibiotalar joint center to the lateral malleolus. The anterior-posterior axis of the tibia was defined as the vector resulting from the cross product of the proximal-distal axis and the vector from the tibiotalar joint center to Gerdy’s tubercle. The coordinate system of the fibula was created by translating the coordinate system of the tibia, as defined at 0° flexion with no applied loads, from the tibiotalar joint center to the location of the lateral malleolus.

The six DOF path of passive plantarflexion-dorsiflexion of the tibiotalar joint of the intact ankle was established from 10° dorsiflexion to 30° plantarflexion. Throughout the range of motion, the positions that satisfied the condition of zero forces and moments across the joint were determined as the path of passive plantarflexion-dorsiflexion. The reference position for the intact ankle state was defined at 0° flexion with zero external applied forces or moments by the robotic testing system.

A 5 Nm inversion moment was applied to the intact ankle at 0° flexion, 15° and 30° plantarflexion, and 10° dorsiflexion and the resulting tibiofibular motion was recorded using the optical tracking system [27, 28]. The AITFL was then sharply transected with a scalpel and the loading condition was repeated at each joint position and the resulting tibiofibular motion recorded using the optical tracking system. Extreme care was made not to disrupt the calcaneofibular ligament and anterior talofibular ligament during transection. The PITFL was then sharply transected under direct visualization and the IOM was transected to 10 cm above the tibiotalar joint line. The loading condition was again repeated at each joint position and tibiofibular motion recorded using the optical tracking system. The orientation and location of the predrilled screw hole was then confirmed with a guide wire and a 3.5 mm cannulated tricortical screw was placed manually over the guide wire from lateral to medial to achieve syndesmotic fixation. The loading condition was again repeated at each joint position and tibiofibular motion recorded using the optical tracking system. Finally, the tricortical screw was removed and a fiber-wire suture was threaded through the same drill hole from lateral to medial and secured to the medial surface of the tibia with an endobutton. The fiberwire was then secured to the fibula over a suture button. Tensioning of the suture button fixation was achieved with a spring scale at a force of 20 N in an effort to standardize fixation as was done in a previous study [31]. The loading condition was again repeated at each joint position and tibiofibular motion recorded using the optical tracking system.

Outcome measures included tibiofibular medial-lateral translation, anterior-posterior translation, and external rotation in response to each applied moment and flexion angle in the following joint states: 1) intact ankle, 2) AITFL transected (isolated AITFL injury), 3) AITFL, PITFL, and IOM transected (complete injury), 4) 3.5 mm cannulated tricortical screw fixation, and 5) suture button fixation. All outcome measures were reported relative to the reference position of the intact ankle as initially defined. The suture button data set for one ankle was not included in analysis due to inadvertent manipulation of the optical markers during testing. Repeated measures analysis of variance with a Bonferroni correction was performed to compare the differences in tibiofibular motion between both injury states and repair techniques and the intact ankle at each flexion angle. Significance was set at a *p*-value of < 0.05. A power analysis was completed prior to the start of this study based on preliminary testing data and determined that nine specimens would be required to achieve appropriate statistical power.

## Results

### Syndesmotic injury

**Fig. 2:**
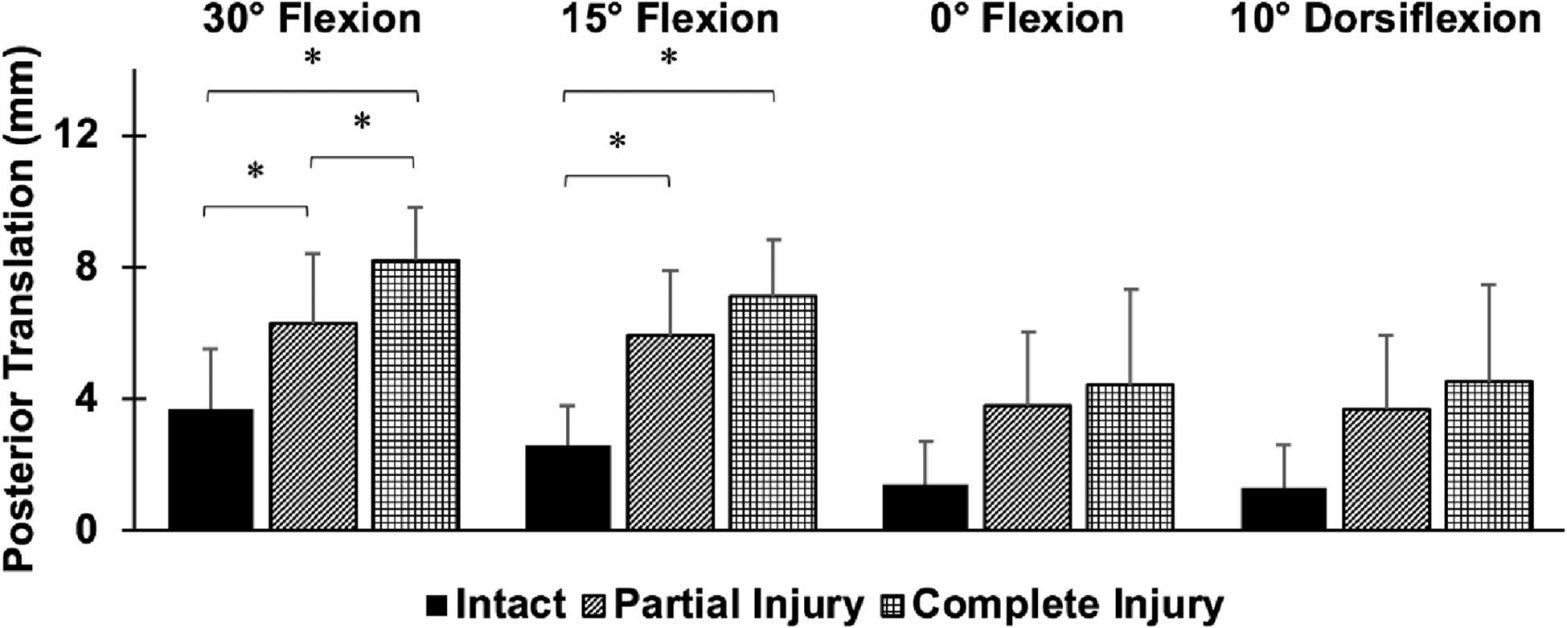
Posterior translation of the fibula relative to the tibia (mean ± SD; 9 specimens) in response to a 5 Nm inversion moment at 30° and 15° plantarflexion, 0° flexion, and 10° dorsiflexion for (1) the intact ankle, (2) the AITFL transected ankle, and (3) the completely injured ankle. *p < 0.05

**Fig. 3:**
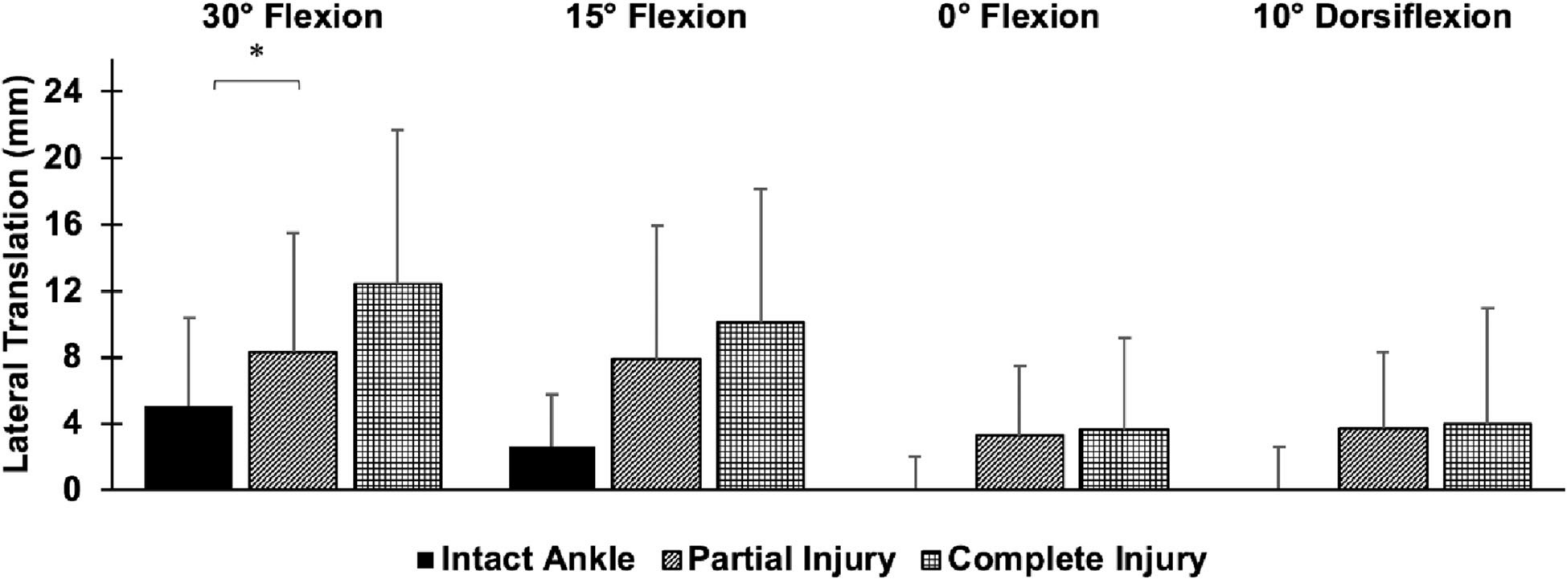
Lateral translation of the fibula relative to the tibia (mean ± SD; 9 specimens) in response to a 5 Nm inversion moment at 30° and 15° plantarflexion, 0° flexion, and 10° dorsiflexion for (1) the intact ankle, (2) the AITFL transected ankle, and (3) the completely injured ankle. *p < 0.05

Posterior translation of the fibula was significantly higher after isolated AITFL injury injury and complete injury compared to the intact ankle at 30° and 15° plantarflexion (*p* < 0.05) (Fig. 2). After isolated AITFL syndesmosis injury, the fibula translated 2.6 mm and 3.3 mm more posteriorly than in the intact state with an inversion moment at 30° and 15° plantarflexion, respectively (*p* < 0.05). The fibula translated 4.5 mm more posteriorly after complete injury than in the intact state with an inversion moment at both 30° and 15° plantarflexion (*p* < 0.05). There was 30% more posterior translation and 50% more lateral translation of the fibula after complete injury compared to isolated AITFL injury at 30° plantarflexion (*p* < 0.05). However, differences between the isolated AITFL and complete injury were not significant in any other ankle positions or directions of motion. While the lateral translation of the fibula increased by 3–4 mm after isolated AITFL syndesmosis injury and 3–7 mm after complete injury compared to the intact ankle in response to an inversion moment, these differences were not statistically significant (*p* > 0.05) (Fig. 3). No significant differences between each injury state and the intact state with respect to internal-external rotation of the fibula were found.

### Syndesmotic repair

**Fig. 4:**
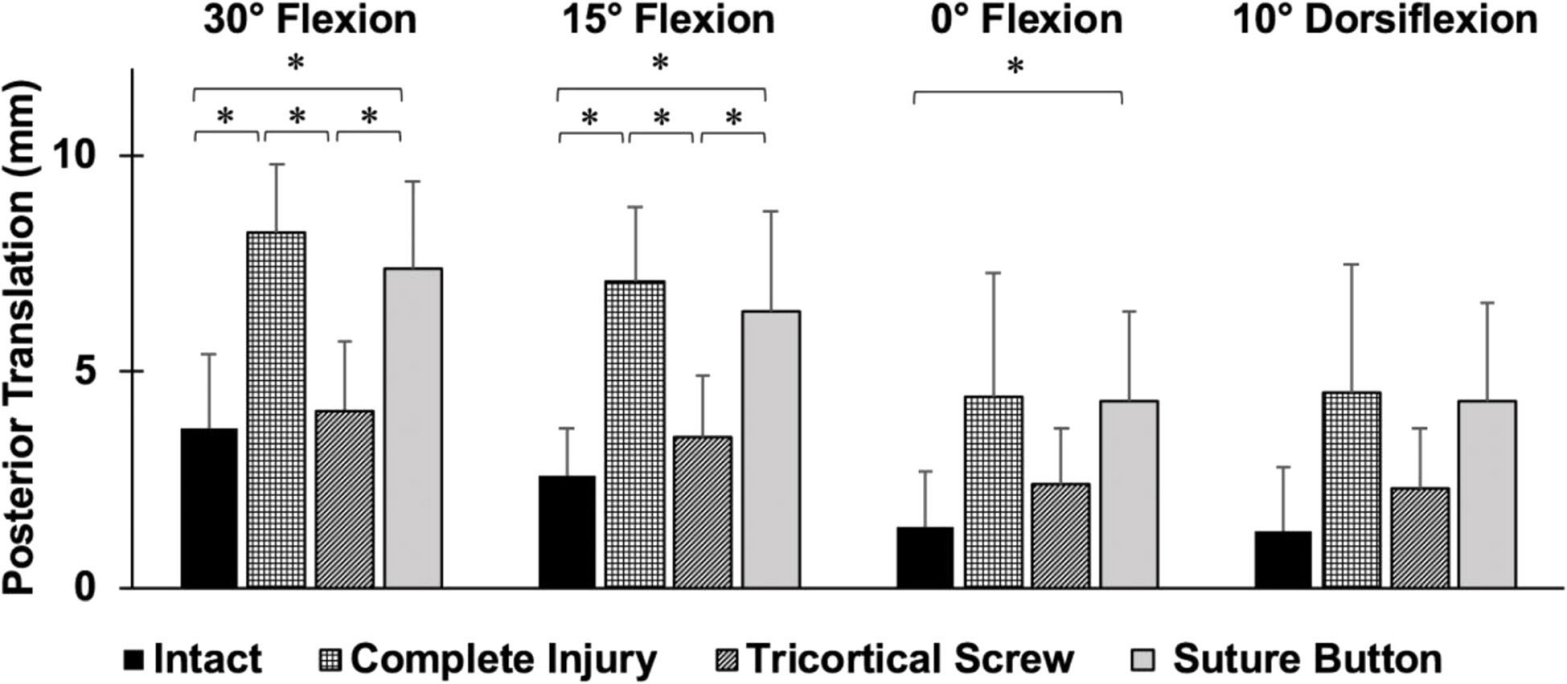
Posterior translation of the fibula relative to the tibia (mean ± SD; 8 specimens) in response to a 5 Nm inversion moment at 0° flexion, 15° and 30° plantarflexion, and 10° dorsiflexion in different ankle states (intact state, complete injury, tricortical screw fixation using a 3.5 mm screw, and suture button repair). *p < 0.05

Significantly higher posterior translation of the fibula with suture button repair was found compared to the intact ankle at 30° and 15° plantarflexion, and 0° flexion (*p* < 0.05) (Fig. 4). The posterior translation of the fibula was 3.7 mm, 3.8 mm, and 2.9 mm more than the intact ankle after suture button repair at 30° and 15° plantarflexion, and 0° flexion (*p* < 0.05). The tricortical screw fixation resulted in significantly less posterior translation compared to the complete injury and suture button repair at 30° and 15° plantarflexion (*p* < 0.05). The tricortical screw had no significant differences compared to the intact ankle. No significant differences between either fixation state and the intact ankle with respect to external rotation and medial-lateral translation were found.

## Discussion

The most important findings of the present study were that both isolated AITFL and complete syndesmosis injury significantly increased posterior translation of the fibula and suture button repair resulted in persistent syndesmotic instability (statistically significant greater posterior translation) compared to the intact ankle in response to inversion, especially in plantarflexion. Isolated AITFL and complete syndesmosis injury resulted in a similar amount of biomechanical ankle instability in all ankle positions except 30° plantarflexion. Contrary to the hypothesis, significant increases in lateral translation of the fibula were not seen after syndesmotic injury in response to inversion torque. Tricortial screw fixation was able to restore motion comparable to the intact ankle in all planes of motion without measurable overconstraint.

Overall, the primary plane of translation following isolated AITFL and complete syndesmosis injury was in the sagittal plane with minimal differences between the two injury states. A previous study showed similar results with regards to fibular motion after syndesmotic injury by showing that the AITFL contributes to fibular stability in terms of sagittal translation and transverse rotation in particular when an external rotation moment is applied in a neutral position [4]. Additionally, the AITFL has been shown to contribute to coronal stability in response to an inversion moment [28]. However, this is the first study to show that isolated injury of the AITFL produces sagittal instability in response to an inversion moment. Previous studies have also demonstrated that suture button repair does not restore sagittal plane stability of the syndesmosis in response to external rotation moment [6, 9, 16, 28]. This study shows that suture button repair does not restore sagittal plane stability of the syndesmosis in response to an inversion moment as well.

The findings of this study suggest that while an external rotation mechanism is usually the cause of syndesmosis injuries, syndesmosis stability in response to an inversion load is important to assess.

In contrast to a previous biomechanical study that demonstrated significant differences in lateral displacement of the fibula following syndesmotic injuries in response to an inversion moment, the increases in lateral translation after isolated AITFL and complete syndesmosis injury in this study were not statistically significant. However, the differences in lateral translation after isolated AITFL (3–4 mm) and complete syndesmosis injury (3–7 mm) compared to the intact ankle are actually larger than those reported in that previous study (2–3 mm) and are clinically relevant [28]. Lateral translation of the fibula in response to an inversion moment after syndesmotic injury is an expected finding given that the talus presses against the lateral malleolus when the ankle is inverted. The data in this study suggests that lateral translation of the fibula is important in the assessment of syndesmotic injury, but also that posterior translation of the fibula may be a more reliable and consistent predictor of the degree of instability that can be evaluated intraoperatively.

This study has implications for decision making for surgical treatment of syndesmotic injuries. Based on this data, assessment of syndesmotic injuries may need to involve evaluation of tibiofibular stability in the sagittal plane in addition to the coronal plane, especially in positions of plantarflexion with inversion. Given that a significant difference between an isolated AIFTL and complete injury was only seen at one ankle position, surgical management may be considered for a West Point Ankle Grade II injury (isolated AITFL injury). When considering surgical fixation methods, it is important to restore native tibiofibular kinematics without overconstraint [2]. Based on the findings of this study, intraoperative assessment of anterior-posterior translation after the use of suture button fixation, especially in ankle positions of plantarflexion with inversion, is critical to ensure that native kinematics have been restored. This is especially important to consider in patients undergoing early rehabilitation and early return to sport, given that single suture button fixation may predispose to reinjury of the syndesmosis with lateral inversion ankle injuries, which are more common than syndesmosis extrenal rotation injuries [5, 17].

While the results of this study suggest that suture button fixation may result in worse clinical outcomes since it was unable to fully restore tibiofibular kinematics in the sagittal plane, recent systematic reviews of randomized controlled trials have shown that suture button fixation has superior outcomes and less complication [7, 10]. Several factors may be contributing to the difference in these kinematic and clinical results. First, the instability seen after suture button fixation in this study may be clinically inconsequential due to the effect of ligamentous healing in the early post-operative period. Second, this instability is seen in a condition with no weight bearing in this study and the addition of weight bearing has been shown to decrease posterior translation of the fibula in the sagittal plane [20]. Thus, under physiological conditions any posterior translation of the fibula may decrease and not be a significant source of instability.

The experimental setup used for this study that combined a six degree-of-freedom robotic testing system and a motion capture system has many unique features. This study utilized a model that assessed tibiofibular kinematics while maintaining in-situ conditions by utilizing a full length fibula and minimizing soft tissue dissection to preserve the proximal tibiofibular articulation. This experimental setup provided a high repeatability of the measurements recorded and allowed for motion of the ankle without constraint of the foot, which was a restraint in serveral previous studies [4, 9, 19].

One of the limitations of this study is that the age of the specimens used for robotic testing is higher than those of the population of patients that typically experience these syndesmotic injuries. Since the tissue properties of a specimen may be altered with age, a thorough evaluation of each specimen prior to testing ensured that there were no gross degenerative change or excessive ligamentous laxity at baseline. Additionally, the effect of the natural healing response in the setting of isolated AITFL injuries cannot be assessed as is a limitation with cadaveric studies. While this study focuses on evaluation of the syndesmosis after injury and repair, it does not provide information regarding the effect of weight bearing on the kinematics of the injury and fixation states, which could further influence treatment decisions. Future studies can utilize the same novel experimental setup with the addition of a compressive load to evaluate these kinematic changes.

## Conclusion

Ankle instability is similar after both isolated AITFL and complete syndesmosis injury and persists after suture button fixation in the sagittal plane in response an inversion stress. Sagittal instability with ankle inversion should be considered when treating patients with isolated AITFL syndesmosis injuries and after suture button fixation.

## Abbrebiations

AITFL: Anterior-inferior tibiofibular ligament
PITFL: Posterior-inferior tibiofibular ligament
IOM: Interosseous membrane
DOF: Degree-of-freedom
UFS: Universal force-moment sensor
